# Care of Infants in the Past: Bridging evolutionary anthropological and bioarchaeological approaches

**DOI:** 10.1017/ehs.2020.46

**Published:** 2020-09-16

**Authors:** Siân Halcrow, Ruth Warren, Geoff Kushnick, April Nowell

**Affiliations:** 1 Department of Anatomy, University of Otago, New Zealand; 2 School of Archaeology and Anthropology, Research School of Humanities and the Arts, ANU College of Arts and Social Sciences, Australian National University, Australia; 3 Department of Anthropology, University of Victoria, Canada

**Keywords:** Infant care, maternal and infant health, bioarchaeology of care, human evolution, palaeoanthropology

## Abstract

The importance of care of infants and children in palaeoanthropological and human behavioural ecological research on the evolution of our species is evident in the diversity of research on human development, alloparental care, and learning and social interaction. There has been a recent surge of interest in modelling the social implications of care provision for people with serious disabilities in bioarchaeology. However, there is a lack of acknowledgement of infant and child care in bioarchaeology, despite the significant labour and resources that are required, and the implications this has for health outcomes within societies. Drawing on the recent proliferation of studies on infancy and childhood in evolutionary anthropology and bioarchaeology, this paper presents ways the subdisciplines may draw on research developments from each field to advance a more holistic understanding of the evolutionary, social and health significance of infant and children care in the past.

## Graphical abstract

**Figure:**
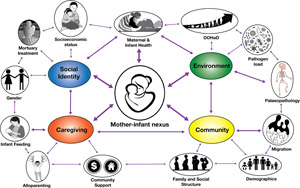

**Media summary:** A tool for evolutionary anthropology and bioarchaeology to produce a holistic understanding of infant care in the past.

## 1. Introduction

Over the past 15 years or so there has been a proliferation of research on infants and children in the bioarchaeological and human evolutionary context (Halcrow & Tayles, 2010; Lewis, 2007; Nowell, 2016, 2020). However, there is a disconnect between evolutionary biological anthropology and bioarchaeological investigations of childhood, despite the complimentary approaches and theoretical developments adopted in these subfields. Like Lancy's (2012) pictorial representation of the house of the Anthropology of Childhood, the rooms between the approaches, instead of being in an open plan architectural design, they have separate rooms with their doors firmly shut. As Lancy (2012: 3) states:

The building might house the Anthropology of Childhood but it is ephemeral as all one sees are the separate doors/cubicles of the more narrowly focused enterprises with no interconnections. The best evidence I can offer for this claim is the rarity of cross-citations and very brief, shallow literature reviews in much of the published work – past and present.

Although there is limited communication between evolutionary anthropology and bioarchaeological approaches to childhood, the subfields have much in common. Clearly, there is a shared interest within the study of palaeoanthropology and human behavioural ecology and bioarchaeology. These fields all examine past childhoods and human experience from an anthropological perspective. Social aspects of care, alloparenting (care by someone other than the biological parent) and infant health are central to evolutionary anthropological and bioarchaeological reconstructions of the human life course (Halcrow, 2020; Hrdy, 2009; Nowell, 2016, 2020; Sear, 2015). In bioarchaeology, infants and children are largely assessed from a palaeopathological context, with little investigation of care (Halcrow, 2020; Le Roy & Murphy, 2020; Tilley & Nystrom, 2019). However, the exploration of care is essential for interpreting the lived experience for infants from the past (Halcrow, 2020; Powell et al., 2016). This paper presents a review of research on infants and children in bioarchaeology and evolutionary anthropology and how we might start to open these closed doors between the approaches.

**Figure 1.**
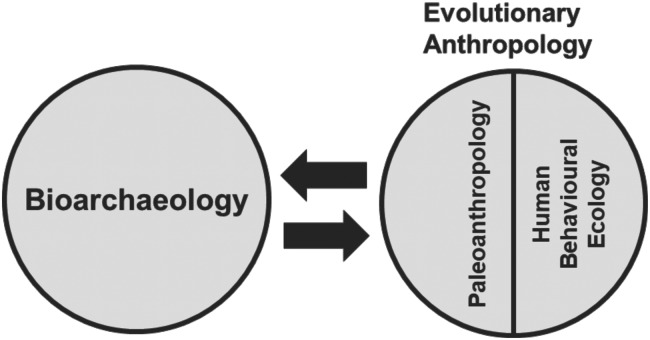
Schematic of relationship between bioarchaeology and evolutionary anthropology.

We review theoretical approaches of care of infants and children used in bioarchaeology and evolutionary anthropology, and highlight the contributions both subfields can make to a holistic appreciation of infant and child care within populations from the recent and deep evolutionary past. Within this review we present a comprehensive bioarchaeological theoretical model for the interpretation of evidence for infant and child care in the past, and demonstrate how this model can be extended with insights from human evolutionary (palaeoanthropological and human behavioural ecology) theory and research (Figure 1). This model assesses the social and health impacts that infant care provision and the mother–infant nexus have on past societies. It considers variables including maternal and infant health and mortality, infant feeding practices, fertility, family and social structure, and population size that may be useful for palaeoanthropologists to consider in their work. We further discuss how the evolutionary anthropological literature may be incorporated into current theoretical and analytical approaches for bioarchaeology. Here we show how bioarchaeological investigations of infant care can benefit from insights from human evolution to extend the model of infant care, including the consideration of the development of human infancy and childhood through the lens of life history theory, alloparental care, energetics, neurobehavioural development of shared intentionality, and human behavioural ecology theories of the relationship between parenting, subsistence and property inheritance.

## 2. Infant and childhood bioarchaeology

Infant and child bioarchaeology, by its very simple definition, is the study of infant and child human remains from the archaeological record (Halcrow & Tayles, 2008; Halcrow & Ward, 2018; Lewis, 2007). Following Buikstra's (1977) definition, the field of bioarchaeology is particularly interested in the study of the human biological consequences of environmental and social factors, through the investigation of elements such as health, disease, mortality and growth.

### 2.1. Brief history of infant and child bioarchaeology

Biological anthropology has had an interest in infants and children since its early beginnings with Boas’ (1912) inquiries into human variation in the growth of children in response to different social and health environments. However, biological anthropologists were mainly interested in the measurement of bodies and skeletal remains, in particular craniometry. Infant and child crania were deemed useless in this endeavour because they were often found disarticulated in archaeological contexts as a result of their unossified joints. It was not until the 1960s that there began to be a focus on infant and child health experience within archaeological research. Francis Johnston was a pioneer in the bioarchaeological study of infants and children, investigating growth and development and mortality from the Indian Knoll skeletal remains (Johnston, 1962). However, it has only been for the past 15 years, approximately, that there has been consistent work in the field, including the development of social theoretical approaches to studying childhood in the past (e.g. Gowland, 2020; Inglis & Halcrow, 2018).

To a large extent, the development of childhood archaeology was precipitated by the rise of feminist critiques within anthropology and archaeology throughout the 1970s, which led to a heightened awareness of aspects of gender and age identity in the past (Halcrow & Tayles, 2008; Lewis, 2007). Around 20 years ago, Kamp (2001), Lillehammer (1989, 2015) and others asked ‘where have all the children gone’ in archaeology (Kamp, 2001), and called for the inclusion of children in archaeological interpretations.

Since the mid-1990s there has been a rapid increase in the amount of research on children and childhood in the past from anthropological, archaeological and bioarchaeological perspectives (Baxter, 2005; Crawford et al., 2018; Lillehammer, 2015; Mays et al., 2017), and the start of the recognition of the value of assessing the young and the intricate relationship between the mother and infant (Blake, 2017; Gowland & Halcrow, 2020; Halcrow et al., 2017; Le Roy & Murphy, 2020). The recognition of the wealth of information that can be gleaned from the study of infants and children has resulted in a large number of bioarchaeological studies investigating mortality, palaeopathology, growth and growth disruption (Lewis, 2007, 2017; Halcrow & Tayles, 2008). Recent advances in social bioarchaeological theory, including an integration of life-course theory and the developmental origins of health and disease (DOHaD) hypothesis, highlight the pivotal place of early life stages in understanding past societies, and the intricate relationship between maternal and child health (Agarwal & Glencross, 2011; Halcrow & Ward, 2017; Gowland, 2020; Gowland & Halcrow, 2020; Gowland & Knüsel, 2006; Sofaer, 2006).

Despite the developments in the field of childhood archaeology and bioarchaeology (Inglis & Halcrow, 2018; Lillehammer, 2015; Mays et al., 2017), there is still a lack of consideration of social aspects of the human experience within bioarchaeology, especially around infant and child care (Halcrow, 2020). Bioarchaeology, by definition, has a bias towards the study of the physical or corporeal body, such as an assessment of manifestations of disease, so the subfield is understandably focused on these biological data (Halcrow & Tayles, 2010).

There has been a recent surge in modelling the implications of care provisioning for people with serious disabilities in the bioarchaeological and palaeopathological literature (Tilley, 2015; Tilley & Oxenham 2011; Tilley & Schrenk 2017). This work has provided essential consideration of social responses and compassion towards people with long-term care needs from disabilities and other health-related conditions. However, Tilley (2015: 100) restricts her definition to health-related care provision, arguing:

some of the skill sets used in health-related caregiving undeniably overlap with some of those employed in assisting healthy women around pregnancy and in raising dependent infants … Nevertheless, caring for an individual with a specific, continuing disability entails qualitatively different sets of actions and motivations, and this is illustrated by comparing non-pathology-related maternal and infant care requirements … This is not to deny that some pregnant women, some mothers and probably many infants may be candidates for healthcare at various times. However, to explain health-related care provision as an extension of infant nurturing is to ignore the quite different, and perhaps more complex, cognitive demands involved in caring for an individual suffering the impacts of disease.

By viewing infant care as nurturing, and not as complex nor requiring the same cognitive demands as health-related care ignores a significant part of the picture of past societies and health-related care (Halcrow, 2020). As noted, bioarchaeology understandably has a focus on the physical manifestations of abnormal bone changes from pathology, and therefore disability. However, here we use a more holistic definition of care and caregiving based on the anthropological work on care and gender by Cancian and Oliker (1999: 2):

a feeling of affection and responsibility combines with actions that provide responsibility for an individual's personal needs or well-being, in a face-to-face relationship. Caregiving includes physical care, such as bathing or feeding a person, as well as emotional care, such as tender touch, supportive talk, empathy, and affection.

There are many reasons for the acknowledgement that care of infants in past societies is crucial to our interpretations of human experience in the past. Western thought discerns medical care as sophisticated and complex, but regards mothering and caring for infants as unskilled, where mothering is seen as ‘instinctual’. The assumption that infant care is unskilled ignores the significant care and resources that are invested in infant and child care and the health effects that this has on mothers and children, and undermines the very complex nature of infant care, including learning to breastfeed (Palmquist, 2020; Tomori et al., 2017). This investment in care is even more significant when we recognise that human infants are born in the most immature (altricial) state of all primates, as is well recognised in human evolution research. Human infants demand 24 hour care, including breastfeeding, help with eating, toileting and regulating temperature, and a significant amount of holding and carrying (Halcrow, 2020). In some past societies, the number of infants and children would have been considerable, and required significant labour and resources within society. Infants and children also carry a high disease burden in societies (World Health Organization, 2019), so their health and care would have been a significant preoccupation of parents and caregivers in the past (Halcrow, 2020).

### 2.2. The significance of infant and childhood care on life in the past

The act of caring for infants and children has repercussions on the adult and child caregivers in society, and the type of care has a direct effect on infant health and wellbeing (Halcrow, 2020; Powell et al., 2016). Young infants require significant care, yet an acknowledgement of the role of care for infants is lacking in the bioarchaeological literature (Halcrow, 2020). Possibly the biggest determinant of foetal and infant health is the care and support they receive, and this starts before birth with the care of the mother (Gowland, 2015; Halcrow, 2020). Foetal and maternal health is arguably the most sensitive measure of population health, given the increased energetic requirements of pregnant and lactating mothers, and the energy requirements for fast-growing foetuses (Altmann & Samuels, 1992; Bogin & Smith, 1996). The effects of malnutrition and many infections can be exacerbated during pregnancy. For example, infection with *Plasmodium vivax* or *Plasmodium falciparum* during pregnancy leads to chronic anaemia and placental malaria infection, reducing the birth weight and increasing the risk of neonatal death (Brabin et al., 2004). The pivotal role of care as a determinant of maternal and infant health is further illustrated if we consider the DOHaD hypothesis (Barker & Osmond, 1986). This considers how environmental impacts on a mother induce physiological changes in foetal and child growth and development that have long-term consequences on later health and disease risk. Furthermore, human infants have underdeveloped immune systems coupled with fast growth rates, resulting in their vulnerability to environmental stress (Halcrow & Tayles, 2008; Lewis, 2007).

**Figure 2.**
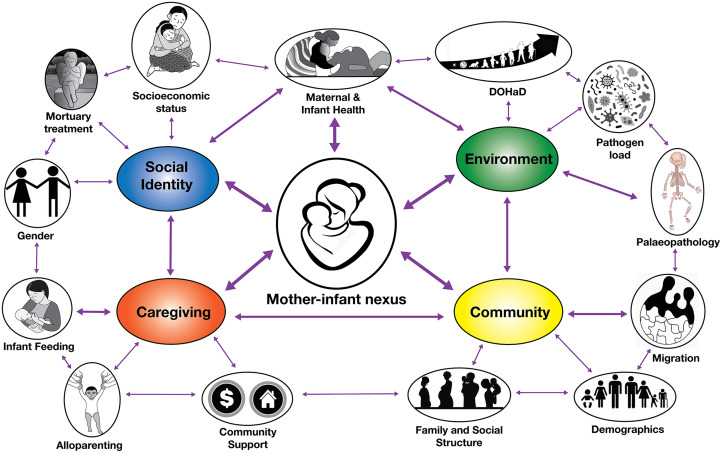
Bioarchaeological model of infant and childcare in the past.

Tilley provides a multiple-step system within her model of care to determine whether people in the past needed long-term health-related care and to interpret the nature of that care within a biosocial context (Tilley & Oxenham, 2011). Presenting a case study of an individual with palaeopathological evidence for quadriplegia in prehistoric Vietnam, Tilley and Oxenham (2011) argue that the survival and good mental health of a person with a serious disability necessitated the provision of long-term, skilled and consistent care, probably involving multiple group members, including the allocation of food/a special diet, water, shelter, bedding, a hazard-free environment, and help with eating and drinking and managing hygiene (removal of wastes, bathing). Interestingly, if we consider human infant care, infants have the same requirements as an individual with a significant disability, and in addition require breastfeeding, special preparation of foods (such as the pre-mastication of food), holding, carrying, rocking, sleeping, massaging and assistance to keep cool/warm (Halcrow, 2020). The recent model proposed for considering the impact of care for infants and children in the past considers multiple factors of past societies (Halcrow, 2020). This model (Figure 2) considers, in addition to the environmental factors that Tilley's model proposes, evidence for infant and maternal health and mortality within past populations, specific diseases through the investigation of pathology (i.e. genetic diseases, and chronic illness from within specific environmental contexts), infant feeding practices (bottle feeding, introduction of solids), demographic information (e.g. fertility and maternal health, size and number of children in families and the community), and family and social structure (through the investigation of migration, gender relationships and health) (Halcrow, 2020). Further extension of this model can be gained through the consideration of evolutionary anthropological approaches that are discussed in Section 4.

## 3. Childhood and evolutionary anthropology

Palaeoanthropology is the study of prehistoric hominins and their primate relatives, and assesses the fossil record within an evolutionary context. The study of juvenile fossil remains has always been central to palaeoanthropological investigations, as they are recognised as being crucial for understanding ontogeny and the evolution of our species (Bogin & Smith, 1996; 2012; Bogin et al., 2018; Bolter et al., 2020). As such, there is not a dedicated ‘palaeoanthropology of infancy and childhood’ such as what developed in bioarchaeology in response to the neglect of research on this subject. The topic of care of infants and social learning also takes a central place in palaeoanthropological investigation of the evolution of our species (Högberg & Gärdenfors, 2015; Hrdy, 2009; Nowell & Kurki, 2020). Human behavioural ecology analyses human behaviour and cultural diversity through the principles of evolutionary theory, and life histories of humans within an ecological and adaptive context (Nettle et al. 2013). Palaeoanthropology and human behavioural ecology draw on an impressive variety of studies to understand the early life course and what makes us human, including using developmental biology, neurobiological development and behaviour, ethnographic, history and primate studies of care to model the past (Bogin & Smith, 1996; Hrdy, 2009; Konner, 2005, 2010).

### 3.1. Brief history of infant and child palaeoanthropology

Palaeoanthropological research is hampered by a rare and fragmentary fossil record. It is hardly surprising, therefore, that spanning over 7 million years of hominin history, only 200–300 infant and juvenile fossil hominin individuals have been discovered (Nowell & Kurki, 2020), very few of which represent early modern humans. The scant nature of the juvenile fossil evidence for these early species limits a comprehensive study of ontogenetic development of hominin evolution, but that does not sideline the importance of these rare discoveries and the contributions they have made to the field. The Taung Child, the type specimen for *Australopithicus africanus*, was integral to the debate that Africa was the cradle of humankind (Tobias, 1984). The recent discovery of a 3.3 million-year-old *Australopithecus afarensis* juvenile from Dikika, Ethiopia, represents the earliest and most complete partial skeleton of a child ever found (Alemseged et al., 2006). Analysis of this juvenile is illuminating our understanding of the growth and development of *Australopithecus afarensis* (Alemseged et al., 2006). The Mojokerto child has been central to the assessment of brain growth in *Homo erectus* (Simpson et al., 2008; Cofran & DeSilva, 2015), and new research on several fossils excavated from Drimolen cave includes a skull fragment believed to belong to a *Homo erectus* child, who was two or three years old at the time of death (Herries et al., 2020). A more substantial number of Neanderthal foetuses, infants and children have been excavated, providing sufficient evidence to investigate Neanderthal growth and development. Interdisciplinary analyses of these fossils enable palaeoanthropologists to gain insight into the complex interactions of growth and organisation of an evolving brain, the birthing process, social systems, weaning, diet, energetics and locomotion (Nowell & Kurki, 2020).

### 3.2. Palaeoanthropology, care and social development

The care of infants and children is central to palaeoanthropological understandings of the evolution of our species (DeSilva, 2011; Foley, 1995; Hrdy, 2009; Piantadosi & Kidd, 2016; Nowell & Kurki, 2020). The investigation of infant care and survivorship is paramount to the human evolutionary context. During hominin evolution there has been increased encephalisation, the birth of more altricial babies, and related to this, the development of alloparenting and cooperative care (Foley, 1995; Hrdy, 2009; Sear, 2015). Nowell and Kurki (2020: 182) recently provided a review of the evolutionary history of infant care and the centrality of its consideration in the evolution of human experience and behaviour. Human infants are unique because they are born with the smallest brain size in proportion to adult size, and across the hominin lineage the absolute brain size in neonates increases over time. These authors draw on Cofran and DeSilva's (2015: 41) observations that the evolution of the human brain enables, but also requires, the cultural capacities (such as food procurement and cooperation) to energetically sustain it. This is shown by Piantadosi and Kidd (2016), who propose a model of how natural selection for large brains may lead to premature and particularly helpless newborns. Caring for these infants and children requires more intelligence and therefore even larger brains. The dynamics can be self-reinforcing and lead to selection for higher intelligence and helpless newborns (Piantadosi & Kidd, 2016).

Evolutionary trends of growth and development can inform us of caregiver–infant interactions and aspects of their social systems. DeSilva (2011) argues that more physically immature, and therefore less mobile, infants would have necessitated greater parental involvement and energetics in their transportation (Hosfield, 2020). Carrying a relatively large infant both pre- and postnatally has important ramifications for birthing strategies and locomotion (DeSilva, 2011). The loss of body hair probably started with Australopiths (Dávid-Barrett & Dunbar, 2016), and a more upright posture would have necessitated a change in the position of infant carrying from the back to the arms, allowing for more interaction between infants and their caregivers and the development of shared intentionality (Dávid-Barrett & Dunbar, 2016), something that is unique to humans (Tomasello, 2014; Tomasello & Carpenter, 2007). Shared intentionality refers to a suite of social-cognitive and social-motivational skills (e.g. gaze following and joint attention, and social manipulation) that result in collaborative interactions where people share psychological states (Tomasello & Carpenter, 2007).

The development of shared intentionality was an important component in the development of the complex social structure required for the care of human babies. Research in developmental psychology shows the importance of social interaction as a developmental mechanism that infants use to support their understanding of intentionality through interactions with social partners (Brandone et al., 2020). Hrdy (2009) argues that during human evolution it was those infants who were proficient with ascertaining intentionality who were selected for and that this social interaction was central for the development of alloparenting. Alloparenting helps mothers conserve energy, stay better nourished and remain safer, leading to their increased survival and greater reproductive success and evolutionary fitness, despite the long human developmental phase (Bogin & Smith, 1996; Hrdy, 2009). Although alloparenting is not unique to humans, in non-human primates this is normally carried out by juvenile females (Smith, 2010), while for humans, alloparenting is done by a wider range of people in the community, including fathers and grandparents (Hrdy, 2009; Palmquist, 2020).

Recent work by Nowell (2016) has highlighted the importance of play in human neurodevelopment, where the extended period of development in humans allows additional years to learn, transmit, practise and modify social behaviours. Symbolic material cultural studies are used alongside psychological and neurobiological research to understand cognitive development in the past (Nowell, 2016). Nowell (2016: 95) states ‘the key to understanding their cognitive abilities lies not in absolute brain size or encephalization quotients but rather in our ability to reconstruct their childhoods and to use the biological and archaeological evidence to piece together how they spent that time’.

## 4. Opening the doors between bioarchaeology and evolutionary anthropology in the ephemeral house of the anthropology of childhood

What can bioarchaeology draw from palaeoanthropology and human behavioural ecology in the study of care in the past?

### 4.1. Fitness costs and benefits of care

Evolutionary anthropological approaches recognise that, although there are many evolutionary forces shaping human bodies and behaviour, natural selection is central in explaining adaptations (Winterhalder & Smith, 2000). Evolutionary anthropology approaches, therefore, view parenting adaptations as biological features that serve the inclusive-fitness interests of parents. Although a given parental behaviour may be in the best interest of the offspring, parental and offspring fitness interests are not always perfectly aligned (Parker et al., 2002). Evolutionary ecology models of parenting rest on the assumption that parental actions incur fitness benefits and costs, and thus parents should behave in ways that optimise this trade-off (Borgerhoff Mulder, 1992). The problem for translating this set of tools to bioarchaeological and palaeontological contexts is that the benefits and costs are difficult, if not impossible, to study in these settings. By looking at coarser-grained differences, the evolutionary anthropological approach may be useful. For instance, we might expect very different parenting strategies in societies where care-independent sources of mortality are higher (owing to, for instance, the presence of epidemics or other disease-related factors), compared with those where they are lower. Increases in care-independent sources of child mortality lead to a non-linear decrease in the fitness-maximizing level of parental effort (Harpending et al., 1990; Quinlan, 2007). For example, Quinlan (2007) studied the effect of extrinsic (care-independent) mortality risk on parental behaviour using comparative evidence from an existing cross-cultural dataset and specific evidence from ethnographic work. He found a curvilinear relationship between various population-level measures of parental care (sleeping proximity, age at weaning, and others) and proxy measures of extrinsic mortality risk including warfare, and pathogen load. With low to moderate levels of increasing environmental risk, parental care increased, but further increases in environmental risk led to decreases in parental care. Quinlan (2007) argues that this supports the theory that humans show reduced parental effort in environments where parenting cannot improve offspring survival. He found similar effects studying changes in extrinsic mortality and parental care over a span of 75 years in a rural community in Dominica (Quinlan, 2010).

### 4.2. Life history growth, energetics and trade-offs near the start of life

As discussed, palaeoanthropological studies have highlighted that humans have extremely fast foetal-like postnatal growth compared with other hominins and non-human primates. We argue that it is important for bioarchaeologists to consider developmental biological phases of the early life course in the interpretations of their health data. A consideration of human early developmental life stages is encapsulated within Life History Theory. This model, originating from biology, has been subsequently developed and applied to evolutionary anthropology by Hill (1993), McDade (2003) and others. This is a branch of evolutionary biology that explains variation in human developmental rates and mortality across the life cycle stages of gestation, infancy, childhood, adolescence and adulthood (Hill, 1993; McDade, 2003). Within this model a main tenet is that resources are limited, and invested in three primary areas of growth, maintenance and reproduction, and that resources allocated for one purpose cannot also be used for another, thus making trade-offs inevitable. During different developmental stages there are varying demands on resources, e.g. during infancy there are a lot of resources put into growth and development of immunity. This is highly relevant to infant and child bioarchaeology because the risk of infection and death from infectious disease is dramatically elevated in the first year of life; the energetic demands of infancy are extreme and trade-offs can be expected to be especially severe, particularly in low-resource settings; immune deficits following under-nutrition early in life have been shown to persist for weeks, and in some cases years; and it is likely that exposure to infectious disease early in life has long-term consequences for immune function (Bourke et al., 2016). We also argue that this model could be extended by integrating evidence for cultural and social determinants (e.g. food allocation and therefore nutrition) in the context of the social life-course approach. For example, a study that uses a social life-course approach to investigate dietary isotopes during childhood (i.e. cultural factors that could influence weaning and food allocation at different parts of the life course), could be greatly enhanced by the life history approach. Here an integration of the life history approach takes into account the energy (and therefore food) demands for children at different ages, and also other constraints on infants including when potential pathogens are introduced with the introduction of non-maternal food resources at a time of significant immune development (Inglis & Halcrow, 2018).

### 4.3. Alloparenting

The importance of alloparenting for our species and evolutionary history may be useful to consider in interpretations of maternal and infant and child care in bioarchaeology. Human maternal energy expended during pregnancy and lactation is significant (Altmann & Samuels, 1992). Alloparenting is argued to be selected for, at least in part, in response to the energetic demands of a developmental life phase, potentially causing significant energetic burden in a mother for several years (Hosfield, 2020). For humans, as mentioned, alloparenting in humans is done by a wider range of individuals in the community, such as siblings, fathers and grandparents (Hrdy, 2009; Mace & Sear, 2005; Palmquist, 2020). The African proverb ‘it takes a village to raise a child’ is particularly apt when considering past care practices. The loss of this village could be extended to the loss of cultural and social support that we see in many Western societies today and in the past (Cunningham, 2005), which can have detrimental effects on mothers and the care of their children, and therefore their health and wellbeing. Similarly, we can consider in certain social contexts in the past, where insufficient resources and support led to the disintegration of alloparenting within families. For example, Hodson and Gowland (2020) found in a study in post-medieval London that when maternal resources were low, infants had significant growth stunting and pathological indicators on their skeletons. In these cases of impoverishment, babies and young children were often left with older siblings who may not have had the means to adequately care for them, or left by themselves, while their mothers and fathers were forced to work in low-paid jobs (Hodson & Gowland, 2020).

### 4.4. Shared intentionality

Shared intentionality may be useful for bioarchaeologists to consider, particularly during the early years of life, which is a period of significant neuro-behavioural development. Tomasello and Carpenter (2007) argue for the important role of shared intentionality in theories of human development. They highlight the infant gaze leading to joint attention, and social manipulation leading to communication. This is seen as central to the evolution of mother–infant communication when the parent requires cryptic information about the offspring's ‘need’ (Kushnick, 2009). There may be some level of manipulation of the parent by the offspring, but this can be mitigated when there is a fitness cost to a response to infant crying (Kushnick, 2009).

There is notable variation cross-culturally and among social groups in adult–infant communication patterns. For example, although some WEIRD (Western, educated, industrialised, rich and democratic) societies have a focus on communication with infants through gaze (Akhtar & Gernsbacher, 2008), others have far more kinesthetic and tactile communication through close body contact (Akhtar & Gernsbacher, 2008). Touch, posture and vocal cues are argued to be important cues to intentions (Akhtar & Gernsbacher, 2008), and therefore central in communication development. For infants, touch is the first sensory modality to develop (Montagu, 1986), and is arguably the most important contributor in infant development of communication, attachment and emotional regulation (Cascio et al., 2019; Hertenstein, 2002).

Psychological literature shows that during early childhood, care through feeding (and therefore touch and potentially visual factors) represents a pivotal experience for the development of the relationship and synchrony between mother and child, in which emotional signals promote the communication of needs and desires (Feldman, 2007; Lieberman & Slade, 2000). Through feeding there is a development of close emotional engagement and a ‘conversational’ setting, where parents make sense of their baby's expressiveness and communicate their empathy and understanding, laying the foundations for their future affective and social communication (Feldman, 2007). This is important to consider in bioarchaeology when social contexts may lead to a loss of this interaction for infants and children, leading to psychological and health deficits. An example of this is the structural violence occurring against the poor and destitute in the workhouses during the Great Irish Famine, where infants were ‘nursed out’ of the workhouses by other women for payment of care, or infants were separated from their parents within the institutions (Geber, 2005). This undoubtedly led to emotional suffering for both parents and infant, and potentially psychological deficits and/or poor health outcomes later in life.

### 4.5. Subsistence, fertility, property and parental care

The Neolithic transition is often viewed as an important turning point in the history of human societies, ushering in pervasive changes across the human social landscape, including the way we parent. Human behavioural ecologists have explored the change in parenting in relation to subsistence, and this should be something that is central to consider in the bioarchaeological models of care.

One such factor is the relationship between subsistence and fertility and the implications that can have on care (Halcrow, 2020). Studies of modern foragers, such as !Kung from southern Africa, space their births up to four years (Howell, 2010). In societies that cultivate, on the other hand, interbirth intervals are much shorter (Bentley et al., 1993). Intensive farmers tend to take this to a further extreme (Kramer & Boone, 2002). Lancaster and Lancaster (1987) argued that the transition from ‘low-density’ societies (foragers and extensive farmers) to ‘high-density’ ones (intensive agriculturalists and others) was accompanied by a change in parental considerations within different resource settings, the former requiring the rearing of ‘fit, healthy’ children and the latter requiring that, as well as the provision of resources that are unequally distributed within the community. Within this constrained resource environment, parental values are modified, which can lead to cultural regulation of the number of children reared, infanticide, child neglect and social systems of dowry (Lancaster & Lancaster, 1987).

A further change in society associated with the Neolithic transition was the emergence of property and its inter-generational transition in the form of marriage payments and inheritance (Gurven et al., 2012; Shennan, 2011). Concerns over inheritance can have important ramifications for parental care of infants and young children (Kushnick, 2010). Inheritance patterns with the development of agriculture and child care may be used to understand parenting behaviour in past societies. Recent bioarchaeological research on Chinese Bronze Age Eastern Zhou dynasty infant feeding patterns using chemical analyses of diet indicates gender preference in food allocation (Miller et al., 2020). The findings suggest that feeding children was a significant aspect of socialisation and cultural gendering of individuals (Miller et al. 2020), and that this finding may be related to the social system of patrilineal inheritance in historic and modern-period China.

### 4.6. Technology and childcare

Technologies related to childcare, so important for our way of parenting today, were probably used to some degree by humans in populations studied by bioarchaeologists and palaeoanthropologists. Kushnick (2020) examines how the three major evolutionary approaches to human behaviour – human behavioural ecology, evolutionary psychology and cultural evolutionary approaches – can inform our understanding of the relationship between parenting behaviour and technology. Behavioural ecology approaches emphasise how parents behave in ways that balance the fitness costs and benefits of childcare and competing activities. The introduction of water-lifting devices, for instance, which raise water to the ground surface for usage, has a relatively deep history (Yannopoulos et al., 2015) and may signal something about how children were raised. The introduction of water pumps may reduce maternal energy expenditure and affect birthrate and child-rearing behaviours and health as a consequence (e.g. Gibson & Mace, 2006). Similarly, baby-carrying devices might arise as the result of a woman's need to free her hands for subsistence work. Kushnick (2020) showed that, in an initial analysis of 77 societies from the Standard Cross-Cultural Sample, baby-carrying technology was present in around 75% of societies where women had high levels of responsibility for subsistence work, but only 50% where women had lower levels of responsibility in this domain. Sturdier varieties of carrying devices, such as cradleboards, may leave archaeological traces (Mattori et al., 1987), while cloth-based ones, such as slings, may not. Some use of these devices may leave physical traces on the body such as cranial modification from cradle boarding that has been seen in the bioarchaeological record (Pomeroy et al., 2013).

### 4.7. Maternal health and altriciality from an evolutionary perspective

From an evolutionary perspective, it is interesting that during human pregnancy women can be subject to significant medical complications (Haig, 2019). For instance, postpartum haemorrhage is the leading cause of maternal mortality today (Abrams & Rutherford, 2011). Haig (2019) asks why placentas are less reliable organs than hearts or kidneys and why maternal hearts and kidneys are more subject to catastrophic failures during pregnancy than at other times. Here he argues that pregnancy involves an interaction between genetically distinct individuals whose cooperation is countered by evolutionary conflicts of interest. Abrams and Rutherford (2011) explain the occurrence of postpartum haemorrhage from an evolutionary biology perspective where humans have developed vulnerability to this condition from changes in placental invasiveness and vascular remodelling to counteract gravitational effects of bipedalism. They cite a cross-cultural study of indigenous birth practices where traditional childbirth attendants’ actions (Lefèber & Voorhoeve, 1998) mimic World Health Organization recommendations on cord traction and uterine massage to induce placental birth (Abrams & Rutherford, 2011). Abrams and Rutherford (2011) argue that the cross-cultural occurrence and variability in this practice illustrates the deep evolutionary past of placental vulnerability (Abrams & Rutherford, 2011). It is important to consider the evolutionary perspective on maternal health care and childbirth in bioarchaeological studies. As is shown today, more than one-third of all women who give birth do so without skilled birth assistants, which can lead to poor health outcomes for mother and baby (World Health Organization et al., 2015). Similarly, in resource-poor settings in the past, this probably had detrimental implications for maternal and child health.

The human birthing process with larger neonate skulls and changes in the configuration of the pelvis with bipedalism would have necessitated the development of help during birth (Trevathan, 1987). Various hypotheses have been put forth for the truncation of the hominin gestational period, leading to an extremely altricial infant. The traditional view was that there is an ‘obstetric dilemma’ (OD), where there is a trade-off between a large skull and a constrained pelvic outlet (Washburn, 1960). However, more recent work explores a metabolic hypothesis which examines human maternal energy expenditures, where this is two times greater near the end of pregnancy than pre-pregnancy and a maximum threshold is met (Dunsworth et al., 2012). Wells et al. (2012) have argued that health factors related to ecological and cultural changes, especially with agricultural development, resulted in increases in infectious disease and poorer nutrition and therefore poorer growth outcomes, including the pelvic cavity. However, others have highlighted the modern biomedical approach to pregnancy and birth as leading to the perceived OD. Stone (2016) presents a comprehensive review of cephalopelvic relationships from evolutionary biology, palaeoanthropology, bioarchaeology, medical anthropology and biomedical approaches. She argues that the OD theory shifts the focus away from physiological and cultural components that have evolved in concert with bipedalism that ensure safe birth for the baby and maternal outcome (Stone, 2016). She argues that the medical risk for women today, and we would argue in some historic periods, is tied to biomedical management of birth and the pregnant woman's body, and structural inequalities (Martin, 2001) that women may face today, including poverty, lack of education and poor nutrition.

### 4.8. What can human evolution studies draw from bioarchaeology?

By drawing on bioarchaeological frameworks of care, palaeoanthropologists may gain a deeper appreciation of the health and social repercussions of care practices and the social and cultural environments on mothers, babies and others in the past. Tilley's model of care has been applied to Neanderthal individuals (Spikins et al., 2018), but has not been considered broadly in the context of care for infants and children in palaeoanthropology. As mentioned above, our model to investigate infant and child care in the past considers variables including maternal and infant health (palaeopathology) and mortality, infant feeding practices, fertility, family and social structure, and population size, and supplies a useful framework in this regard (Halcrow, 2020).

Fitting with the nature of palaeoanthropological finds of single or small numbers of individuals, there has been some shift of focus in bioarchaeology and palaeopathology from cemetery population-level evidence for health and disease to detailed contextualised osteobiographies through a Bioarchaeology of Individuals approach to complement population-level analysis (Hosek & Robb, 2019; Stodder & Palkovich, 2012). Social bioarcheological approaches examine health and care through a contextualised approach that considers aspects of identity (age, sex, class, ethnicity) (Gowland & Thompson, 2013), and some of these factors of identity may be applicable to the palaeoanthropological context.

Palaeoanthropology has some focus on palaeopathology (e.g. Trinkaus, 2018). However, there is scope for further work in this area to look at the evolution of disease within populations. It is important to look at children, in particular, as they carry a large disease burden within populations (Lewis, 2007, 2017; Halcrow & Tayles, 2008). Bioarchaeology has a main interest in major transitional events in the natural and cultural environments, such as climate change, the agricultural transition and how this affected past populations (Larsen, 2015; Robbins Schug, 2020). Similarly, it is important to consider climate and social factors such as population demographics in the understanding of pathology and mortality in individuals within the palaeoanthropological context.

Some palaeoanthropological studies in the Upper Palaeolithic already explore aspects of mortuary behaviour to ascertain information on social responses to infant loss, familial relationships and social age (e.g. Einwögerer et al., 2006; Humphrey et al., 2019; Nowell, 2020). Recently, there has been an increased research interest on grief and emotion from the archaeological context, and part of this research is starting to consider community members’ responses to infant and foetal death (e.g. Cannon & Cook, 2015; Le Roy & Murphy, 2020; Murphy, 2011). The purported marginalisation of foetuses along with infants in the archaeological record, including location and simplified mortuary treatment, has led some scholars to interpret that they were of little concern beyond immediate family members (Cannon & Cook, 2015). Consideriation of evidence for intense grief after miscarriage and infant death starts to challenge the notion that their loss was of little consequence (Murphy, 2011).

Evolution anthropology (as well as bioarchaeology) could draw on a multitude of other research on infant care, breastfeeding, sleep, and infant and child socialisation research from social anthropology (including medical anthropology) and social archaeological approaches (Baxter, 2005; Gottlieb, 2004; Han, 2020; Palmquist, 2020; Tomori et al., 2018).

## 5. Conclusion

The investigation of care for infants in the past from multiple lenses offers an opportunity to begin to pry the doors open between evolutionary anthropology and bioarchaeology within Lancy's (2012) Anthropology of Childhood house. Social aspects of infant and maternal care, alloparenting and infant health are central to evolutionary and bioarchaeological reconstructions of the human life course. Through an investigation of the human evolutionary past of the development of child care, and human biological and social development, we can contribute to a more holistic understanding of child care networks in the archaeological context. Similarly, the use of socially nuanced bioarchaeological theoretical frameworks of infant and child care within evolutionary anthropology may extend the understanding of health care and aspects of social structure for the deep past.
